# Fertilizer management and soil type influence grain zinc and iron concentration under contrasting smallholder cropping systems in Zimbabwe

**DOI:** 10.1038/s41598-019-42828-0

**Published:** 2019-04-23

**Authors:** Muneta G. Manzeke, Florence Mtambanengwe, Michael J. Watts, Elliott M. Hamilton, R. Murray Lark, Martin R. Broadley, Paul Mapfumo

**Affiliations:** 1 Soil Fertility Consortium for Southern Africa (SOFECSA), Department of Soil Science and Agricultural Engineering, University of Zimbabwe, Harare, Zimbabwe; 2 Inorganic Geochemistry, Centre for Environmental Geochemistry, British Geological Survey, Nottingham, NG12 5GG United Kingdom; 3 School of Biosciences, University of Nottingham, Sutton Bonington Campus, Leicestershire, LE12 5RD United Kingdom

**Keywords:** Solid Earth sciences, Geochemistry

## Abstract

Micronutrient deficiencies remain prevalent in food systems of southern Africa, although advances in biofortification through crop breeding and agronomy provide opportunities to address these. We determined baseline soil availability of zinc (Zn) and iron (Fe) and the effects of soil type and farmer management on extractable soil Zn and Fe and subsequent concentration in cereal and legume grains under two contrasting agro-ecologies in Zimbabwe. Soil and crop surveys were conducted in Hwedza and Mutasa Districts of Zimbabwe in 2015–16 on 350 locations over different soil types. Fields with different levels of productivity (designated as “most” and “least” productive fields) were sampled using an inherited hierarchical randomized sampling design. Grain Zn and Fe concentration in maize (*Zea mays*), sorghum (*Sorghum bicolor*), finger millet (*Eleusine coracana*) and cowpea (*Vigna unguiculata*) were generally insufficient for adequate human nutrition. A Linear Mixed Effects (LME) model revealed that diethylene triamine penta-acetic acid- (DTPA) extractable soil Zn concentration and grain Zn concentration were affected primarily by field productivity level. DTPA-extractable soil Zn concentration was more than two-fold greater on the most productive fields (mean 0.8 mg kg^−1^) than on the least productive fields, with mean grain Zn concentration of 25.2 mg grain Zn kg^−1^ which was 13% greater than seen on the least productive fields.  An interaction effect of field productivity level and total soil Zn concentration on DTPA-extractable soil Zn concentration suggests potential contribution of organic matter management to unlocking unavailable forms of soil Zn. DTPA-extractable soil Fe and grain Fe concentration were primarily affected by soil type and crop type, respectively. The LME modelling approach revealed additional soil geochemical covariates affected DTPA-extractable soil Zn and Fe concentration and grain Zn and Fe concentration within Districts. Future studies can therefore be powered to detect their roles at wider spatial scales for sustainable management of crop Zn and Fe nutrition.

## Introduction

Globally, the prevalence of micronutrient deficiencies (MNDs) due to inadequate dietary intake remains high. Over 2 billion people are likely to be at risk of inadequate dietary micronutrient intakes, especially of zinc (Zn) and iron (Fe)^1,2^, with greater risks in developing countries^3–5^. In contrast to steady reductions in risk in Latin America, East and South Asia over the past 50 years, Zn and Fe deficiencies have remained high in sub-Saharan Africa (SSA)^4,6^. This is partly attributed to less total food intake and dietary diversity in SSA^2,6^, poorer soil quality, and fewer options for soil fertility management in smallholder systems^7^. A high reliance on plant-based foods containing high levels of anti-nutritional factors such as phytate^8^ also presents a challenge for tackling dietary Zn and Fe intakes in the region.

Most smallholder farming in eastern and southern Africa is predominantly cereal and legume-based. These farming systems typically rely on sub-optimal application of macronutrient- (nitrogen-N, phosphorus-P, potassium-K) containing mineral fertilizers of <10 kg ha^−1^ year^−1^ due to lack of farm-level resources and limited access to fertilizer^9,10^. Research on smallholder cropping systems has often focussed on improving the fertility of poor quality and highly weathered soils for increased crop productivity using integrated soil fertility management (ISFM)^11–13^. Integrated soil fertility management can include: combined applications of mineral NPK fertilizers and locally available organic nutrient resources, legume-cereal rotations/intercrops, the use of appropriate germplasm and good agronomic practices for increased soil and crop productivity. Whilst evidence of increased crop yields with ISFM is well established in SSA^14–16^, there has not been much work on establishing the effects of the environment and farmer management options including soil type, organic matter management and crop choices on grain Zn and Fe concentration.

The effect of baseline soil type on maize (*Zea mays* L.) grain Zn concentration in Malawi was reported by Chilimba *et al*.^17^ and showed that maize grown on vertisols had ~30% greater grain Zn concentration than on other soil types. This effect was attributed to underlying differences in soil mineralogy and not to differences in fertilizer use or soil management strategies. For a person consuming 300 g maize day^−1^, this would translate to differences of ~1.5 mg Zn intake between soil types. Adult women consuming maize in proximity to vertisols had a median Zn intake of 6.4 mg person^−1^ day^−1^, while those near non-vertisol acid soils had a median Zn intake of 4.8 mg person^−1^ day^−1^ ^18,19^, which was consistent with predictions based on baseline soil/grain surveys^17^. These studies demonstrated that variations in inherent micronutrient levels in different soil types may have implications on human nutrition.

Previously, we showed that application of organic nutrient resources can increase maize grain Zn concentration and dietary Zn supply in legume and cereal- based cropping systems in Zimbabwe, compared with the use of mineral NPK-based fertilisers alone^7,20^. The application of organic manures also increased biomass and grain yield, translating to more animal feed and greater purchasing power, which could also help to alleviate MNDs.

Baseline geospatial information on soils and cropping systems is likely to be useful for optimizing and evaluating current genetic and agronomic biofortification strategies employed to combat MNDs^2,7,20–24^. However, the heterogeneity of farming systems and resources within and across farms still presents major challenges to understanding factors governing crop nutritional quality in southern Africa. The aims of this study were: (i) to determine the effect of variation of total and extractable soil Zn and Fe concentration on the Zn and Fe concentration of grains of maize, finger millet (*Eleusine coracana* (L.) Gaertn.), sorghum (*Sorghum bicolor* (L.) Moench) and cowpea, under contrasting agro-ecologies, (ii) to determine the effects of soil organic matter (SOM) management on availability of soil Zn and Fe to growing plants; and iii) to explore soil factors which underpin variation in extractable soil Zn and Fe and grain Zn and Fe concentration across farms. We hypothesize that: 1. Clay soils and most productive fields have larger values of extractable soil Zn and Fe concentration, and grain Zn and Fe concentration, than sandy soils and least productive fields and; 2. There are various soil geochemical factors governing extractable soil Zn and Fe concentration and grain Zn and Fe concentration.

## Methods

### Study sites

The study was conducted in Hwedza District (18°41′S, 31°42′E; 1380 m.a.s.l.) and Honde Valley, Mutasa District (18°35′S, 32°45′E; 912 m.a.s.l.) in Zimbabwe during the 2015–16 pre-cropping season period (October to November) and the cropping season (December to May). The Districts were selected on the basis of their contrasting agro-ecologies, which we then used as a basis to assess the availability of micronutrients in soils^25,26^. Agro-zonation in Zimbabwe is defined in terms of mean annual rainfall during a unimodal season that occurs between November and April, with Natural Region (NR) I receiving the highest annual rainfall of >1000 mm annum^−1^ and NR V receiving ≤ 450 mm annum^−1^ ^25,26^. Hwedza encompasses three of Zimbabwe’s agro-ecological regions, NR) II to IV, receiving 450–800 mm year^−1^. Soils in this community are broadly classified as Lixisols^27^ with pockets of Luvisols^28,29^. Maize is the dominant crop under a mixed crop-livestock farming system^30^.

**Figure 1:**
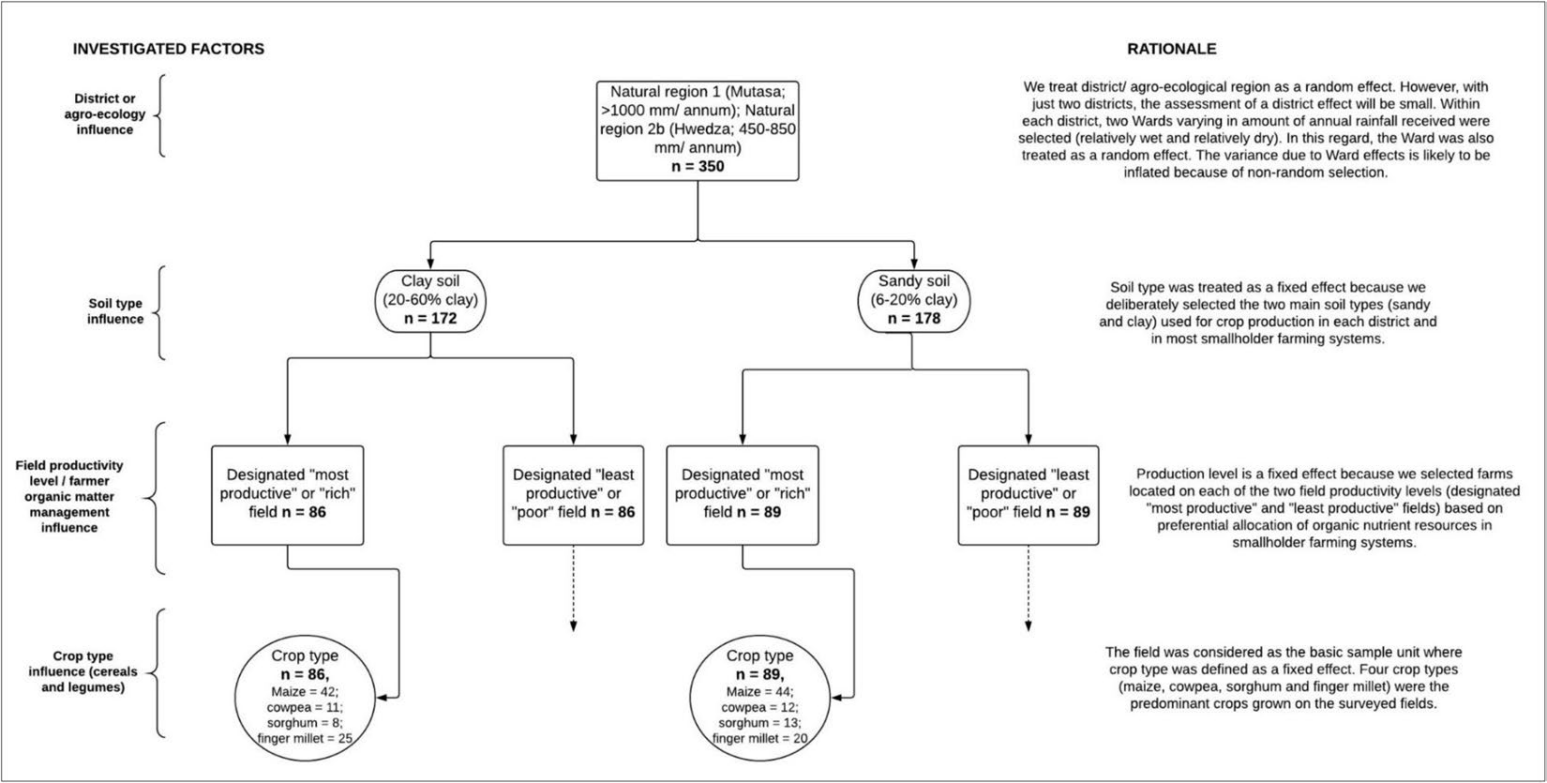
Nested sampling design employed in Hwedza and Mutasa to assess effects of soil type, field productivity level and crop type on DTPA-extractable soil Zn and Fe concentration and grain Zn and Fe concentration.

Honde Valley extends from the eastern border of Zimbabwe into Mozambique with an average altitude of 900 m, rising to above 1800 m. The area experiences hot and humid weather from late October to end of April and hot summers averaging 30 °C during the dry months of the year. Mean annual rainfall is >1000 mm year^−1^ falling mostly between October and May, although the valley often receives some precipitation throughout the year, making it the wettest part of the country. Soils in this area are broadly classified as Acrisols and Ferralsols with patches of Lixisols and Arenosols^27,29^. The main food crops grown in Honde Valley are maize and groundnut (*Arachis hypogaea* L.). Owing to the terrain and high rainfall which do not favour cattle rearing, few farmers own cattle with the majority using mineral fertilizer only in crop production. The favourable soils and climate (high rainfall and high temperature) of Honde Valley results in most smallholder farmers being contracted by various private companies to grow cash crops such as banana (*Musa acuminata* Colla) and chilli pepper (*Capsicum annuum* L.) for export. Specialized and diversified farming of plantation crops such as tea (*Camellia sinensis* L.), coffee (*Coffea arabica* L.), and macadamia nut (*Macadamia integrifolia* Maiden) is also done by surrounding large-scale farmers. During the entire 2015–16 cropping season, Hwedza received 627 mm year^−1^ and Mutasa received 1183 mm year^−1^ (Fig. 1; Supplementary Material).

### Soil and crop sampling survey

#### Factors considered during soil and crop surveys

The study comprised a soil survey conducted during the dry months of October and early November before the onset of rains for the 2015–16 cropping season (December to April 2016), and a grain survey conducted during harvesting time in March/April 2016. The soil and crop surveys were conducted using an inherited sampling design where the Soil Fertility Consortium for Southern Africa (SOFECSA) has been conducting work on ISFM and climate change adaptation options for alleviating food insecurity and malnutrition^11,15,31^. The surveys were conducted in Dendenyore (agro-ecological zone/NR II) and Ushe (agro-ecological zone/NR III-IV) Wards in Hwedza, and Mandeya Ward 30 (agro-ecological zone/NR I) and Sahumani Ward 8 (agro-ecological zone/NR II) in Honde Valley, Mutasa. Research approval for this study was obtained from the Department of Agricultural Technical and Extension Services (AGRITEX) of The Government of Zimbabwe’s Ministry of Lands, Agriculture and Rural Resettlement.

Co-ordinated soil and grain samples were collected from two sites at each of 175 farms using a nested sampling design (Fig. 1). The factors considered to influence plant-availability of soil Zn and Fe concentration and grain Zn and Fe concentration were agro-ecology (defined by variations in the amount of rainfall received within a particular area per annum), study site, soil type, field productivity level and crop type within a particular field. We therefore expected variation in soil Zn and Fe availability and uptake between the two study sites to emanate from variation in soil moisture^1^. Informed by previous studies^1,17,20^, soil type was taken into consideration because it is a major factor governing micronutrient availability in soils. The soil fertility status and productivity potential of a field on smallholder farms is strongly influenced by farmer preference of nutrient resource allocation and management at a field-level^32,33^. In this regard, most productive fields are often allocated higher quantities of organic and mineral fertilizers compared with least productive fields. We therefore expected farmer soil fertility management options to affect plant-availability of Zn and Fe as well as grain Zn and Fe concentration.

#### Selection of farms for soil and crop sampling

Using village lists provided by Agricultural Extension Workers (AEWs) in each District, we identified farms located on clayey (20–60% clay) and sandy (6–20% clay) soil types which represent the major soils used for crop production and excluded farms on multiple soil types and/or on other soil types. From farms identified within each soil type, we then selected 178 fields (n = 89 farms) located on sandy soils and 172 fields (n = 86 farms) on clayey soils (Fig. 1) using independent random sampling. To allocate fields into these two major soil textural classes, our fields selection criteria was guided by AEW’s knowledge of the study sites. The soil textural classes were subsequently confirmed through laboratory analyses. On each target farm, two fields were then selected on the basis of their productivity level (described by farmers as “most” and “least” productive fields, or simply “rich” and “poor” fields, respectively)^32^. Farmer’s knowledge of their farm and local diagnostic indicators guided selection of designated “rich” and “poor” fields (see Table 1; Supplementary Material)^31–37^. Information on the management of these fields, including fertilizer type and rates applied to each field during the previous (2014–15) cropping season as well as the resource group (RG) of each host farmer was obtained from the field owners. According to Mtambanengwe and Mapfumo^32^ and Zingore *et al*.^33^, smallholder farmers broadly fall into three distinct resource groups based on their resource endowments, including farm-level physical resources and access to crop production inputs, which in turn, influence their nutrient resource allocation patterns to different fields and crops. Accordingly, resource-endowed (RG1) farmers often have large livestock herds and access to capital to purchase mineral fertilizers hence apply high levels of nutrients to both rich and poor fields. Intermediate (RG2) and resource-constrained (RG3) farmers often fail to produce good grain yields due their low financial and nutrient resource-base^32,33^.

All sampled fields were georeferenced using a hand-held geographical positioning system (GPS) unit (GPS72, Garmin, Kansas City, USA). Within each field, a single composite soil sample was obtained after sampling 10 points on a “W” transect at a depth of 0–20 cm using either a ½” sand or Edelman combination auger (W signature series, Eijkelkamp, American Falls, USA), depending on soil texture. Soil samples were air-dried, sieved through a 2-mm stainless steel sieve and ground to <40 µm in an agate Retsch PM400 Planetary Ball Mill (Haan, Germany). For each of the selected fields (typically 0.45–1.50 ha) for maize and (0.05–0.40 ha) for cowpea, sorghum and finger millet; grain yield was quantified at physiological maturity from three replicate 9 m^2^ plots within each field pooled to produce composite grain samples. Fresh weight for the harvested composite grain sample was recorded. About 5 maize ears and ~500 g for each of cowpea, sorghum and finger millet were collected from each plot, air-dried, and processed for grain yield quantification at 12% (maize) and 9.5% (cowpea, finger millet, sorghum) moisture content. A subsample of ~100 g of the processed grain was milled through a 0.5 mm diameter stainless steel sieve (Thomas-Wiley Model 4 Laboratory mill, Thomas Scientific, Swedesboro, New Jersey, USA) for subsequent elemental analysis of Zn and Fe. Genotypic variation in Zn and Fe uptake was not considered within this study.

### Soil and grain analysis

Extractable soil Zn and Fe were measured using the diethylene triamine penta-acetic acid (DTPA) extraction method^38,39^ to represent the fraction of Zn and Fe potentially available for plant uptake. Mineral analyses were conducted using inductively coupled plasma-mass spectrometry (ICP-MS; Agilent 8900 Triple Quad, Santa Clara, USA). Each batch of 40 samples included two reagent blank samples, three random sample duplicates and three Certified Reference Materials (CRMs) for quality control. The CRMs used were BGS 102 (Ironstone soil, British Geological Survey-NERC, Nottingham, UK), NIST 2710a (Montana 1 soil, US Geological Survey, National Institute of Standards and Technology, Virginia, USA) and IRMM-443 (Euro soil, Institute of Reference Materials and Measurements, Geel, Belgium). Soil pH was determined using 0.01 *M* calcium chloride (CaCl_2_) solution. To confirm field dignosis of soil type, the hydrometer method for measuring soil texture by Gee and Bauder^40^ was followed using seived soils. There was consistence between field and laboratory diagnosis of soil type. The sandy soil category contained soils with a sand and clay content ranging from 60–90% and 6–20%, respectively and the clay soils had between 20–40% sand and 20–60% clay content, respectively. Using the finely milled (Ø < 40 µm) soil sample, SOM and total elemental concentration were analysed using the loss-on-ignition (LOI) and mixed acid solution (HF 2.5 mL:HNO_3_ 2 mL:HClO_4_ 1 mL:H_2_O_2_ 2.5 mL) methods respectively, as described in Joy *et al*.^41^.

Grain, CRMs (NIST 1573a Tomato leaf; National Institute of Standards and Technology, Virginia, USA; and NIST 1567b Wheat flour; National Institute of Standards and Technology) and blanks were analyzed for total Zn and Fe using the Aqua Regia (2 mL 50% HCl: 5 mL 25% HNO_3_) method and absorbance measured using an Atomic Absorption Spectrophotometer (AAS) (Varian SpectrAA 50, California, USA). Final Zn and Fe concentrations in grain were converted to mg kg^−1^ dry weight (DW).

### Data analyses

The analysis of the data from nested sampling was based on a linear mixed effects (LME) model using the *nlme* package for the R statistical platform^42,43^. Using this model, we treated soil type, field productivity level and crop type as fixed effects because we deliberately chose two soil types from which to select farms at random, and similarly, deliberately chose one field from each of the two defined field productivity levels which are predominant in smallholder farming systems. Overall, we defined crop type grown on each of the two field production levels as a fixed effect. The Districts, Wards within Districts, and farms within Wards were treated as random effects. The field was thus considered the basic sample unit. Summary statistics were computed for the data using R version 3.4.3 (R Core Team^43^). Based on the asymmetric distribution of the residuals, data were log transformed before any analyses. Box plots were plotted using SigmaPlot (Systat Software, San Jose, CA) to show distribution of DTPA-extractable Zn and Fe concentration and grain Zn and Fe between study sites, soil types and productivity levels. Using the LME model, we treated soil (type), field productivity level and crop type as fixed effects. Soil type was treated as a fixed effect because it was an operational decision to include sites from each of two soil types from each District. Within the LME model, farms within Wards and Wards within Districts were treated as random effects. Random effects are sources of variation which contribute to the variation of our data rather than through the investigator’s decision. The contribution of a random effect to observed variation is quantified by its variance component, an additive component of the natural variation of the target variable, and it may be informative to compare the variance components for different random effects. Influence of fixed effects (soil type and field productivity level) on DTPA-extractable soil Zn and Fe concentration, and grain Zn and Fe concentration (soil type, field productivity level and crop type), were tested by the analysis of variance (ANOVA) in the *nlme* package. In this analysis, the strength of evidence for the fixed effect is judged by the ratio of the mean square for the fixed effect (e.g. between soil types) to the residual mean square at the level of the analysis at which that fixed effect is replicated. Under the null hypothesis of no effect, the expected value of this ratio is one. A larger ratio is evidence against that null hypothesis, and we obtain a *P* -value, which is the probability of obtaining a variance ratio as large or large if the null hypothesis were true. For a given variance ratio, the *P-*value depends on the numerator degrees of freedom (which describes the complexity of the fixed effect) and the denominator degrees of freedom (which indicates the precision with which the residual mean square is estimated, and depends on the sample size). The possibility of interactions of soil type and field productivity level were considered and tested to assess whether effects of field productivity level or organic matter management on DTPA-extractable soil Zn and Fe concentration and grain Zn and Fe concentration is dependent or independent on which soil type a farm is located on. Possible effects of covariates (soil pH, SOM, total soil Zn and Fe concentration) on DTPA-extractable Zn and Fe concentration and grain Zn and Fe concentration (i.e. DTPA-extractable Zn and DTPA-extractable Fe concentration as additional covariates) were also considered.

The variance component for District in the LME model was used to compare variation in DTPA-extractable soil Zn and Fe concentration in soils from the two Districts and how it compared with the Ward and farm variance components. Mean, range, median and standard deviation (SD) values for soil variables and grain Zn and Fe concentration of maize, cowpea, sorghum and finger millet are presented throughout the text. Fields with a DTPA-extractable Zn and Fe concentration of <0.5 mg kg^−1^; and 5.0 mg kg^−1 ^^39^ were defined as having low plant-available soil Zn and Fe.

## Results

### DTPA-extractable Zn and Fe concentration in soils in Hwedza and Mutasa

#### Variation in DTPA-extractable Zn and Fe concentration in soils with respect to the random effects

**Figure 2:**
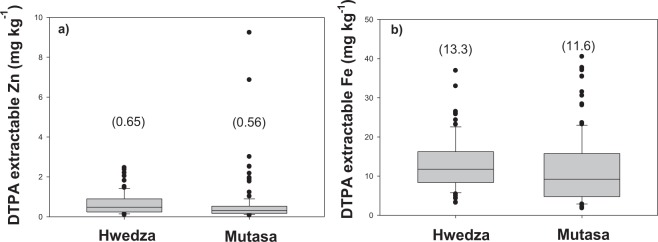
DTPA-extractable soil Zn and Fe concentration in Hwedza and Mutasa Districts. *Boxes* represent interquartile range (IQR) and the *midline* represents the median. *Whiskers* represent largest and smallest concentrations within 1.5*IQR of the box ends. Values in parentheses denote mean DTPA-extractable soil Zn and Fe concentration in each site.

The between-District variance component for DTPA-extractable Zn concentration in soils (0.034; Table 1) was an order of magnitude smaller than the farms within Wards (0.293) or fields within farms (0.351) variance components. The between-Ward variance component of DTPA-extractable Zn concentration in soils was also smaller than the farm within Wards and field within farms variance components. This suggests that farmer soil fertility management options and short-range effects are more important in predicting soil Zn status than broader spatial variation, although the latter can still identify broad spatial trends. In the majority of soils, DTPA-extractable soil Zn concentration was small. DTPA-extractable soil Zn concentration ranged from 0.1–9.2 mg kg^−1^ (mean 0.6 ± 0.06; median 0.3) across sites. Over 51% and 72% of soils had DTPA-extractable Zn concentration of <0.5 mg kg^−1^ in Hwedza and Mutasa, respectively. DTPA-extractable soil Zn concentration ranged from 0.1–2.5 mg kg^−1^ (0.65 ± 0.06 mg kg^−1^, median 0.5 mg kg^−1^) in Hwedza, and 0.1–9.2 mg kg^−1^ (0.56 ± 0.09 mg kg^−1^, median 0.3 mg kg^−1^) in Mutasa (Fig. 2a; Table 2; Supplementary Material).

**Table 1:** Variance components showing influence of soil type and field productivity level on DTPA-extractable soil Zn and Fe concentration.

Model - variance components for DTPA-extractable Zn and Fe		
Soil type and field productivity level effect		
Source	Variance component	
	DTPA-extractable Zn	DTPA-extractable Fe
District	0.034	1.0 × 10^−9^
Ward within District	0.083	0.156
Farm within Ward	0.293	0.192
Field within farm	0.351	0.158

Similarly, the between-District variance component for DTPA-extractable soil Fe concentration was negligible (1.0 × 10^−9^; Table 1), but variance components of comparable magnitude were obtained for the Wards within Districts (variance component = 0.156), farms within Wards (variance component = 0.192) or fields within farms (variance component = 0.158) effects. DTPA-extractable soil Fe concentration was large in both Hwedza (13.3 ± 0.8 mg kg^−1^, median 11.8 mg kg^−1^) and Mutasa (11.6 ± 0.7 mg kg^−1^, median 9.2 mg kg^−1^; Fig. 2b), with only 7% (Hwedza) and 23% (Mutasa) of the soils having DTPA-extractable Fe concentrations below the critical level of 5.0 mg kg^−1^ required for optimal crop growth. DTPA-extractable soil Fe concentrations in Wards within Hwedza (range 3.2–36.9 mg kg^−1^) and Mutasa (range 1.8–40.5 mg kg^−1^) is detailed in Table 2 (Supplementary Material).

Random effects accounted for variations in DTPA-extractable soil Zn and Fe concentration differently. For example, the Ward within District variance component for DTPA-extractable soil Zn (0.083) was smaller than the variance component for farms within Wards and fields within farms random effects (0.293 and 0.351, respectively; Table 1). The three variance components for DTPA-extractable soil Fe concentration were comparable. This suggests that differences between farms and between fields within farms (attributable to farmer management effects) are more important for DTPA-extractable soil Zn than are spatial variations at broader scales within the District, whereas for plant available Fe all these factors make comparable contributions to the variability.

#### Variation in DTPA-extractable soil Zn and Fe concentration with respect to soil type as a fixed effect

**Figure 3:**
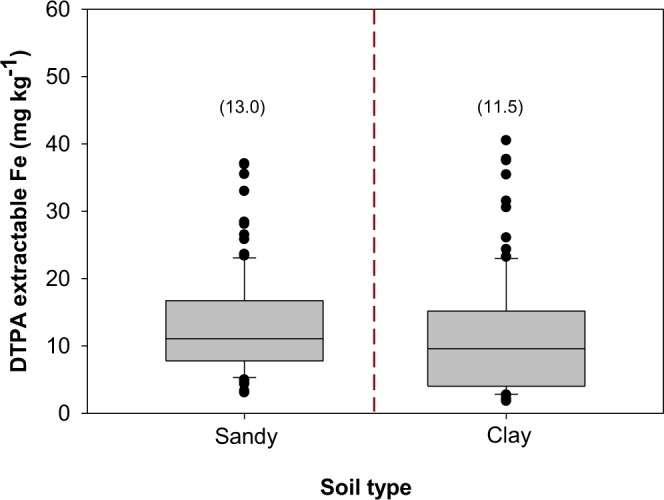
DTPA-extractable soil Fe concentration due to soil type effect across study sites. *Boxes* represent interquartile range (IQR) and the *midline* represents the median. *Whiskers* represent largest and smallest concentrations within 1.5*IQR of the box ends. Values in parentheses denote mean DTPA-extractable soil Fe concentration for sandy and clay soils.

There was no evidence of soil type effect on DTPA-extractable soil Zn concentration (P > 0.05; Table 2). DTPA-extractable soil Zn concentration ranged from 0.07–3.0 mg kg^−1^ (mean 0.5, median 0.3; SD 0.5) on sandy soils and between 0.08 and 9.2 mg kg^−1^ (mean 0.7, median 0.4, SD 1.2) on clay soils (Excel Supplementary File). Significant effects of soil type on DTPA-extractable soil Fe concentration were observed (P < 0.01; Table 2). Across sites, DTPA-extractable Fe concentration ranged from 3.1–37.1 mg kg^−1^ (mean 13.0, median 11.1, SD 7.4) and 1.8–40.5 mg kg^−1^ (mean 11.5, median 9.6, SD 8.7) on sandy and clay soils, respectively (Fig. 3).

**Table 2:** Linear Mixed Effects (LME) model ANOVA output on effects of soil type and field productivity level and soil x field productivity level on DTPA-extractable Zn concentration and grain Zn and Fe concentration in Hwedza and Mutasa.

DTPA-extractable soil Zn concentration	Numerator df	Denominator df	Variance ratio	*P*-value
Soil main effect	1	96	0.978	0.3252
Field productivity level main effect	1	111	48.46	<0.0001
Soil • Field productivity level interaction	1	111	0.079	0.779
**DTPA-extractable soil Fe concentration**				
Soil main effect	1	96	7.853	0.0061
Field productivity level main effect	1	111	0.043	0.8359
Soil • Field productivity level interaction	1	111	0.041	0.8390
**Grain Zn concentration**				
Soil main effect	1	154	1.101	0.2956
Field productivity level main effect	1	178	9.937	0.0019
Crop type main effect	3	178	0.413	0.7442
Soil • Field productivity level interaction	1	178	2.787	0.0968
Soil • Crop type interaction	3	178	1.582	0.1953
Field productivity level • Crop type interaction	3	178	0.180	0.9099
Soil type • Field productivity level • Crop type interaction	3	178	0.867	0.4594
**Grain Fe concentration**				
Soil main effect	1	154	2.276	0.1334
Field productivity level main effect	1	178	0.427	0.5141
Crop type main effect	3	178	104.505	<0.0001
Soil • Field productivity level interaction	1	178	3.831	0.0519
Soil • Crop type interaction	3	178	1.083	0.3577
Field productivity level • Crop type interaction	3	178	2.444	0.0656
Soil type • Field productivity level • Crop type interaction	3	178	0.614	0.6071

df = degrees of freedom, variance ratio = F value from ANOVA output. • indicates interaction between two factors.

#### Variation in DTPA-extractable soil Zn and Fe concentration with respect to field productivity level

**Figure 4:**
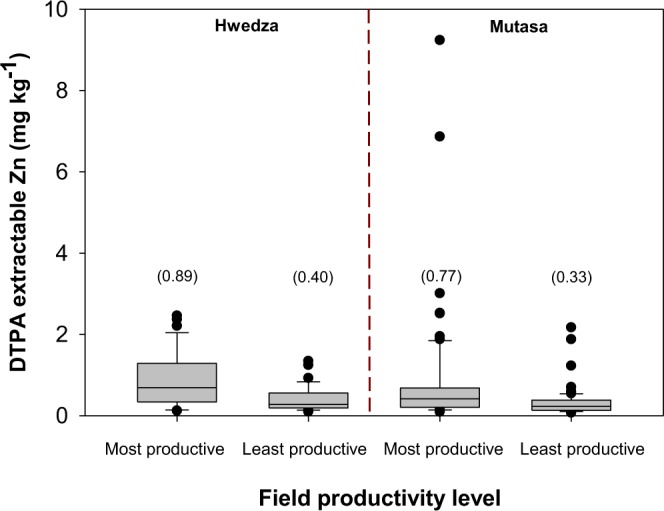
DTPA-extractable soil Zn concentration between most and least productive fields in Hwedza and Mutasa. *Boxes* represent interquartile range (IQR) and the *midline* represents the median. *Whiskers* represent largest and smallest concentrations within 1.5*IQR of the box ends. Values in parentheses represent mean DTPA-extractable soil Zn concentration for most and least productive fields in Hwedza and Mutasa.

Field productivity level effect on DTPA-extractable soil Zn and Fe concentration was treated as a fixed effect within the LME model. There was a significant effect of field productivity level on DTPA-extractable soil Zn concentration (P < 0.0001), but not on DTPA-extractable soil Fe concentration (P > 0.05) (Table 2). Across sites, the most productive fields had larger DTPA-extractable Zn concentration (0.1–9.2 mg kg^−1^, mean = 0.8 ± 0.1, median 0.5, SD 1.2) compared to least productive fields (0.1–2.5 mg kg^−1^, mean = 0.4 ± 0.04, median 0.3, SD 0.4). In Hwedza, the most productive fields had larger mean DTPA-extractable soil Zn concentration of 0.89 ± 0.10 mg kg^−1^ (range 0.1–2.5; median 0.7; Fig. 4) which was more than double a mean DTPA-extractable soil Zn concentration of 0.40 ± 0.05 mg kg^−1^ (range 0.1–1.3; median 0.3; Fig. 4) measured on least productive fields which often receive sub-optimal rates of organic nutrient resources. Similarly, the most productive fields in Mutasa had a mean DTPA-extractable soil Zn concentration of 0.77 ± 0.16 mg kg^−1^ (range 0.1–9.2; median 0.4) compared to a mean DTPA-extractable soil Zn concentration of 0.33 ± 0.04 mg kg^−1^ (range 0.1–2.2; median 0.2) on least productive fields (Fig. 4). This substantial variation in DTPA-extractable soil Zn concentration between the most and least productive fields across study sites is likely to reflect the preferential allocation of organic nutrient resources by farmers increasing plant availability of Zn in soils.

The survey revealed a wide range of NPK fertilizer application rates (Table 1; Supplementary Material) and soil fertility management practices (Fig. 2; Supplementary Material) employed by farmers and this could have had a large effect on DTPA-extractable soil Zn concentration. For example, up to 14 ‘scotch carts’ ha^−1^ of cattle manure (~350–500 kg per scotch cart) and >170 kg mineral N ha^−1^ were applied in some of the most productive fields with between 0 and 5 scotch cart loads applied to least productive fields (Table 1; Supplementary Material). The amount of fertilizer applied varied by farmer resource endowment where resource endowed (RG1) farmers tended to apply the largest amounts of organic nutrient resources and mineral fertilizer to their fields irrespective of productivity level. In addition, nearly 50% of farmers in Hwedza used ISFM options which encompassed combined use of organic nutrient resources (cattle manure, woodland leaf litter and composts) with mineral NPK fertilizers in crop production. In contrast, 83% of farmers in Mutasa used mineral NPK fertilizers while only 8% combined mineral NPK fertilizers with cattle manure (Fig. 2; Supplementary Material). Across sites, a mean DTPA-extractable soil Fe concentration of 12.3 mg kg^−1^ and 12.7 mg kg^−1^ was measured in the most and least productive fields, respectively (Excel Supplementary File). There were no interaction effects of soil type and field productivity level on DTPA-extractable soil Zn and Fe concentration (Table 2a,b).

### Grain yields and grain Zn and Fe concentration in cereals and cowpea

#### Variation in grain yields and grain Zn and Fe concentration of crops grown in Hwedza and Mutasa

Across sites, maize grain yields ranged from 0.1–5.2 t ha^−1^ (mean 1.4 ± 0.05; median 1.3). Average grain yields attained for cowpea were 0.2 ± 0.04 (range 0.04–1.0; median 0.7), for sorghum were 0.6 ± 0.1 (range 0.1–1.4; median 0.8), and for finger millet were 0.3 ± 0.05 (range 0.05–1.5; median 1.5) (Table 3; Supplementary Material).

For grain Zn concentration, the field within farm variance component (variance = 0.060) was two-fold more than the District effect on grain Zn concentration (variance = 0.030; Table 3). Similarly, the field within farm effect on grain Fe concentration variance component (variance = 0.114) was an order of magnitude larger than the District effect (variance = 0.008), Ward within District (variance = 0.002) and farm within Ward (variance = 0.018; Table 3) effects. The District, the Ward within District, and the farm within Ward effects were larger for grain Zn concentration compared to grain Fe concentration (Table 3) indicating potentially stronger effects of agro-ecology on grain Zn than grain Fe concentration.

**Table 3:** Variance components showing influence of soil type, field productivity level and crop type on grain Zn and Fe concentration.

Model - variance components for grain Zn and Fe concentration		
Soil type, field productivity level and crop type effect		
Source	Variance component	
	Grain Zn concentration	Grain Fe concentration
District	0.030	0.008
Ward within District	0.027	0.002
Farm within Ward	0.049	0.018
Field within farm	0.060	0.114

All four crop types had comparable mean grain Zn concentrations of between 22.5 to 24.9 mg kg^−1^ (range 7.9–42.4) (Table 3; Supplementary Material). Across study sites, maize grain Fe concentration ranged from 8–66 mg kg^−1^. Despite smaller grain yields of small grains compared with maize, finger millet had a wide variation in grain Fe concentration of between 25–139 mg kg^−1^ and sorghum had an even wider range of 16–308 mg kg^−1^. Whilst some of the high grain Fe concentration could be due to soil/dust contamination, small grains might have greater capacity to potentially meet dietary Fe requirements of rural households compared with staple maize. Cowpea grain Fe concentration ranged from 18–54 mg kg^−1^ in Hwedza and 32–108 mg kg^−1^ in Mutasa District giving a mean grain Fe concentration of 43.7 mg kg^−1^ (Table 3; Supplementary Material).

#### Soil and crop type effect on grain Zn and Fe concentration

Soil type (defined by “sandy” and “clay” texture class) did not affect grain Zn and Fe concentration significantly (P > 0.05; Table 2c,d). Grain Zn concentration ranged from 7.9–39.2 mg kg^−1^ (mean 22.0, median 21.9, SD 8.0) on sandy soils and 8.3–42.4 mg kg^−1^ (mean 25.7, median 26.6, SD 7.2) on clay soils (Excel Supplementary File). Grain Fe concentration ranged from 7.8–308 mg kg^−1^ (mean 41.5, median 33.8, SD 31.2) on sandy soils and 12.0–139 mg kg^−1^ (mean 45.1, median 35.5, SD 26.5) on clay soils (Excel Supplementary File).

There were no significant differences among the crops in grain Zn concentration (P > 0.05; Table 2). Crop type had a significant effect on grain Fe concentration (P < 0.0001; Table 2). Sorghum had the largest grain Fe concentration of 78.1 mg kg^−1^, followed by finger millet, cowpea and maize with grain Fe concentrations of 62.3, 43.7 and 28.0 mg kg^−1^, respectively (Excel Supplementary File). No significant interaction effects of soil type, field productivity level and crop type on grain Zn and grain Fe concentration were observed (P > 0.05; Tables 2 and 2).

#### Variation in grain Zn and Fe concentration of crops grown on fields varying in productivity level

**Figure 5:**
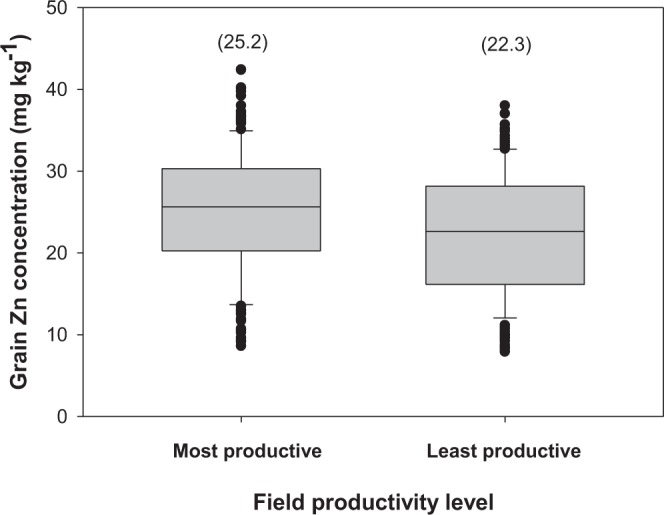
Grain Zn concentration in all crop types with respect to field productivity level in Hwedza and Mutasa. *Boxes* represent interquartile range (IQR) and the *midline* represents the median. *Whiskers* represent largest and smallest concentrations within 1.5*IQR of the box ends. Values in parentheses represent mean grain Zn concentration for most and least productive fields.

Field productivity level had a significant effect on grain Zn concentration (P = 0.002; Table 2). The most productive fields had grain Zn concentration of 25.2 mg kg^−1^ (range 8.6–42.4, median 25.6, SD 7.8) compared to 22.3 ± 0.6 mg kg^−1^ (range 7.9–38.0; median 22.6, SD 7.5) on least productive fields (Fig. 5). In Hwedza, the most productive fields had larger grain Zn concentration of 22.5 mg kg^−1^ (median 24.3; SD 9.7) compared to a mean grain Zn concentration of 16.5 mg kg^−1^ (median 15.5, SD 7.1; P = 0.002) measured on least productive fields. In Mutasa, the most and least productive fields had comparable mean grain Zn concentrations of 25.7 mg kg^−1^ (median 26.0; SD 6.9) and 24.7 mg kg^−1^ (median 25.1; SD 7.3), respectively. These larger variations in grain Zn concentration between most productive fields and least productive fields in Hwedza than in Mutasa could be attributed to additional Zn supplied from frequent use of organic nutrient resources in Hwedza District (Fig. 2; Supplementary Material), which are then preferentially allocated to more productive fields than poor fields. Field productivity level did not affect grain Fe concentration significantly (P > 0.05; see Table 2). The most productive fields had grain Fe concentration of 44.0 mg kg^−1^ (range 12.0–138.9, median 34.5, SD 27.3). The least productive fields had grain Fe concentration of 42.5 mg kg^−1^ (range 7.8–307.7; median 34.1, SD 30.8; Excel Supplementary File).

### Interaction effect of soil factors and covariates on DTPA-extractable soil Zn and Fe concentration, and grain Zn and Fe concentration

#### Interaction effects of soil factors and covariates on DTPA-extractable Zn and Fe concentration

We tested the effects of covariates (soil pH, SOM, total soil Zn and Fe concentration) and their interactions with soil type and field productivity level (as main fixed effects) on DTPA-extractable soil Zn and Fe concentration. DTPA-extractable soil Zn concentration was significantly affected by soil pH (P < 0.0001), SOM content (P = 0.017) and total soil Zn concentration (P = 0.004; Table 4) with soil pH having the largest effect of 0.623 while SOM and total soil Zn concentration had effects of 0.009 and 0.013 respectively, (Excel Supplementary File). Across all sites, there was evidence of larger DTPA-extractable soil Zn concentration at pH ranging between 4.5–5.5, which tended to decline with an increase in soil pH (Excel Supplementary File) which ranged from 4.0–7.3 (see Table 2; Supplementary Material). Increases in SOM content (range 0.3–11.4%) and total soil Zn concentration (range 6.2–193.3 mg kg^−1^; see Table 3; Supplementary Material) were associated with an increase in DTPA-extractable soil Zn. The effect of increase in SOM on DTPA-extractable soil Zn concentration was larger on the most productive fields compared with the least productive fields (as indicated by a larger slope of the regression line on most productive compared to least productive fields).

**Table 4:** Reduced Linear Mixed Effects (LME) ANOVA output of interaction effects of a) field productivity level; b) soil type; c) crop type and covariates on DTPA-extractable soil Zn and Fe concentration and grain Zn and Fe concentration.

Effect	Numerator df	Denominator df	Variance ratio	*P*-value	Effect	Numerator df	Denominator df	Variance ratio	*P*-value
**DTPA-extractable soil Zn concentration**					**DTPA-extractable soil Fe concentration**				
Soil type	1	96	0.912	0.342	Soil type	1	96	9.691	0.002
Field productivity level	1	105	63.04	<0.0001	Field productivity level	1	105	0.045	0.830
pH	1	105	39.73	<0.0001	pH	1	105	32.826	<0.0001
SOM	1	105	5.87	0.017	SOM	1	105	1.439	0.233
Total soil Zn concentration	1	105	8.87	0.004	Total soil Fe concentration	1	105	0.001	0.978
Field productivity level • pH	1	105	0.77	0.381	Soil • pH	1	105	0.394	0.531
Field productivity level • SOM	1	105	0.03	0.866	Soil • SOM	1	105	0.208	0.649
Field productivity level • Total soil Zn concentration	1	105	6.76	0.011	Soil • Total soil Fe concentration	1	105	0.0001	0.991
**Grain Zn concentration**					**Grain Fe concentration**				
Soil type	1	96	4.318	0.072	Soil type	1	96	10.381	0.007
Field productivity level	1	100	22.986	<0.0001	Field productivity level	1	92	2.433	0.151
Crop type	3	100	0.212	0.888	Crop type	3	92	64.136	<0.0001
pH	1	100	4.119	0.039	pH	1	92	1.221	0.217
SOM	1	100	2.161	0.145	SOM	1	92	0.279	0.960
DTPA-Zn	1	100	5.915	0.017	DTPA-Fe	1	92	8.287	0.003
Total soil Zn concentration	1	100	0.253	0.616	Total soil Fe concentration	1	92	0.136	0.644
Field productivity level • pH	1	100	0.008	0.927	Crop type • pH	3	92	3.062	0.588
Field productivity level • SOM	1	100	1.336	0.251	Crop type • SOM	3	92	5.529	0.334
Field productivity level • DTPA-Zn	1	100	2.674	0.105	Crop type • DTPA-Fe	3	92	2.393	0.011
Field productivity level • Total soil Zn concentration	1	100	0.010	0.920	Crop type • Total soil Fe concentration	3	92	2.347	0.156

^•^ indicates interaction between two factors. df = degrees of freedom.

A significant interaction effect of field productivity level and total soil Zn concentration on DTPA-extractable soil Zn concentration was evident (P = 0.011; Table 4). The expected increase in DTPA-extractable Zn for a given increase in total Zn was larger on the most productive fields (effect = 0.013; data not shown) than on least productive fields (effect = −0.008; data not shown).

Inclusion of covariates in the LME model reduces variance components and improves the explanatory capacity of fixed effects. For example, the inclusion of covariates in the LME model had the largest effect on the District variance component resulting in a much smaller variance component attributable to District effects (variance = 0.2 × 10^−6^; Table 5, Model 1), compared with a variance component of 0.034 (with no covariates included; see Table 1). This suggests that the basic soil properties (e.g. soil pH, SOM and total soil Zn concentration) accounted for these broad scale differences in DTPA-extractable soil Zn concentration between contrasting environments, in this case, agro-ecology and may not be useful for predicting differences in DTPA-extractable soil Zn concentration within a District. However, these soil properties could account for broad national scale trends. Soil pH was the only factor which had a significant effect on DTPA-extractable soil Fe concentration (P < 0.0001; effect = −0.380; Table 4).

**Table 5:** Variance components for the reduced Linear Mixed Effects (LME) model on influence of soil type and field productivity level and covariates (soil pH, SOM, total soil Zn and Fe concentration) on DTPA-extractable soil Zn and Fe concentration and grain Zn and Fe concentration (with crop type as an additional fixed effect and DTPA-extractable soil Zn and Fe concentration as additional covariates).

1. Model - variance components for DTPA-extractable soil Zn and Fe concentration		
Reduced model with covariates and field productivity level*total Zn effect		
Source	Variance component	
	DTPA-extractable Zn	DTPA-extractable Fe
District	0.2 × 10^−6^	0.1 × 10^−7^
Ward within district	0.080	0.118
Farm within Ward	0.240	0.147
Field within farm	0.278	0.147
2. **Model - variance components for grain Zn and Fe concentration**		
**Reduced model with covariates effect**		
**Source**	**Grain Zn concentration**	**Grain Fe concentration**
District	0.023	0.001
Ward within district	0.014	4.3 × 10^−10^
Farm within Ward	0.056	0.025
Field within farm	0.057	0.110

#### Interaction effects of soil factors and covariates on grain Zn and Fe concentration

Grain Zn concentration was significantly affected by soil pH (P = 0.039; effect = 0.049) and DTPA-extractable soil Zn concentration (P = 0.017; effect = 0.061) (Table 4). For example, an increase in DTPA-extractable soil Zn concentration resulted in a significant increase in grain Zn. The reduced LME model (model which only includes fixed effects with significant effects on grain Zn or Fe concentration) showed no interaction effects of field productivity level and covariates (soil pH, SOM, total and DTPA-extractable soil Zn concentration) on grain Zn concentration (Table 4).

Grain Fe concentration was significantly affected by DTPA-extractable soil Fe concentration alone (P < 0.05; Table 4). No significant effects of soil pH, SOM and total soil Fe concentration on grain Fe concentration were observed (P > 0.05). On the other hand, inclusion of covariates resulted in soil type having significant (P < 0.01) effects on grain Fe concentration (Table 4) compared to the analysis when covariates were not included (P > 0.05; see Table 2) possibly because of reduced residual variance. When the interaction effects of crop type (the only main fixed effect with significant effects on grain Fe; see Table 2) and covariates on grain Fe concentration were tested, only the crop type and DTPA-extractable soil Fe concentration interaction effect was significant (P < 0.05; effect = 0.007; Table 4).

Inclusion of covariates substantially reduced the Ward within District variance component (variance = 4.3 × 10^−10^; Table 5, Model 2) compared to when no covariates were included (variance = 0.002; see Table 1). This suggests that some of the soil factors (covariates) accounted for variation in grain Fe concentration between Wards within a District. The fields within farms component had the largest effect on grain Fe concentration, with a variance component of 0.110 (Table 5, Model 2). This indicated stronger effects of within-farm management options, in this instance a possible interaction between crop type and DTPA-extractable Fe concentration, on grain Fe concentration compared with agro-ecological effects.

## Discussion

Zinc deficient soils are widespread in Hwedza and Mutasa, where 62% of arable soils surveyed had less than 0.5 mg Zn kg^−1^ required for optimal crop growth^44^. When a Zn limit of 0.8 mg kg^−1^ required for optimal maize growth is considered^39^, the proportion of soils below this threshold was 84%. In contrast, 86% of the soils were above the critical limit of 5.0 mg kg^−1^ DTPA-extractable Fe as reported by Lindsay and Norvell^39^.

DTPA-extractable soil Zn concentration was larger in Hwedza than in Mutasa. These differences could be attributed, in part, to a larger proportion of farmers using ISFM options in Hwedza (31%) than in Mutasa (12%). Plant availability of Zn in cropped soils has previously been found to be influenced by organic matter addition^20^ implying that organic nutrient resources can help to address MNDs in cropping systems through supply of Zn^31^. Interestingly, an interaction effect of field productivity level with total soil Zn concentration was evident. The fixed effect coefficient for the effect of total soil Zn concentration on DTPA-extractable soil Zn was larger on the most productive fields than on least productive fields. This suggests potential contribution of organic matter management to unlocking unavailable forms of Zn into the soil. This however, requires further investigations.

Grain obtained from the most productive fields had a mean Zn concentration of 25.2 mg kg^−1^ compared with 22.3 mg kg^−1^ on the least productive fields and was independent of soil type. The absence of an interaction effect of soil type and field productivity level on DTPA-extractable soil Zn concentration and grain Zn implies the effect of organic matter management on DTPA-extractable soil Zn concentration and grain Zn concentration is consistent across soil types. Thus, if smallholder farmers apply organic nutrient resources to their fields, there is a good chance of increasing the grain Zn nutritive value of their crops. Apart from the crucial role of organic nutrient resources in sustaining maize productivity in southern Africa^16^, organic nutrient resources proved to contribute significantly to DTPA-extractable soil Zn availability as well as grain Zn concentration but not DTPA-extractable soil Fe and grain Fe concentration.

The major drivers of DTPA-extractable soil Fe concentration were soil type and pH. Grain Fe concentration was significantly affected in turn by DTPA-extractable soil Fe concentration, crop type, and their interactions. Some of the dolerite-derived clayey soils from this study had a dark-red colour which indicates oxidized ferric iron oxides which are readily available for plant uptake. Therefore, crops grown under such soils are likely to have greater grain Fe concentration compared to crops grown on sandy soils potentially due to improved soil-crop Fe uptake and/or extrenous dust contamination^18^. On the other hand, availability of Fe in the soil and its subsequent uptake and accumulation in grains depends to a larger extent on soil pH where soil Fe bioavailability and uptake by plants is reduced with an increase in soil pH^1,45^. Adoption of the LME approach which explicity shows effects of farmer management practices on plant and grain Zn concentration and soil geochemical effects on plant and grain Fe concentration enables identification of important baseline drivers of grain quality.

Organic nutrient resources have previously been reported to contribute significantly to extractable fractions of soil Zn^21,31^ and Fe^45^ concentration which might translate to improved grain nutrition. For example, application of mineral N fertilizer increased grain Zn concentration in maize^21^ grown on low-Zn soils compared with treatments which did not receive N fertilizer. Better plant N nutritional status was associated with improved remobilization of Zn from leaves to grains of major cereals^46,47^. This study therefore provides insights for future interventions to promote better Zn nutrition through optimizing N applications in smallholder production systems.

In this study, grain Fe but not grain Zn concentration differed between crop species. Finger millet, sorghum and cowpea had greater grain Fe concentration than maize, as seen previously in legume seeds^48^ and small grain cereals^49^ Overall, grain Zn and Fe concentrations in cereals (maize, sorghum, finger millet) and cowpea were likely to be insufficient for adequate human Zn and Fe nutrition. Maize, which is mostly grown on most productive fields had higher grain yields than the other cereals. Farmer preferential allocation of organic nutrient resources to different fields contributed to variations in crop yields. Smallholder farmers allocate most time and farm resources to the staple maize crop^50,51^, whilst small grain cereals and legumes are typically grown on nutrient-depleted smaller portions of land^52^. Smallholder farmers also have more in-depth knowledge and information on the agronomy of maize production from local extension than for other crops, thus tend to concentrate external nutrient resource application and other critical agronomic paractices such as early planting and timely weeding on maize. This can undermine the productivity and overall contribution to dietary micronutrient supply from other “potentially” nutrient-dense crops. For example, while smallholder farmers do not often apply organic nutrient resources to grain legumes^15,20^ and small grains, our results indicated that these grains gave comparable and/or larger grain Zn concentration than maize. The use of lower-productive fields/practices to grow non-maize crops, with inherently greater nutrition, but risks “lose-lose” in terms of micronutrient supply should be addressed in the context of alleviating MNDs in the global south.

Soil type, field productivity level and crop type accounted for much of the variation in DTPA-extractable soil Zn and Fe concentration, and grain Zn and Fe concentration. Although other soil factors such as soil pH and SOM could have contributed to variations in DTPA-extractable Zn and Fe concentration under smallholder cropping (see Table 4), their inclusion as covariates in the LME model was not useful for predicting differences in DTPA-extractable Zn and Fe at within District scale. Small-range management differences explained most of the spatial variation within Districts. However, soil pH, SOM and variations in total soil Zn and Fe concentration might be useful in accounting for broader-scale nutritional issues rather than contributing to crop Zn and Fe nutrition at the farm-level. A different sampling approach would be useful to assess broader spatial national and/ or regional trends in soil micronutrient distribution. Evidence of soil type effect on DTPA-extractable soil Fe concentration suggests provision of a soils map which shows the distribution of Fe and exploration of alternative options, other than agronomic approaches, which supply dietary Fe requirements of communities at larger risk of deficiency should be explored.

We explored the magnitude by which farmer management might contribute to improved dietary Zn intake at the household level, using data and assumptions from Kumssa *et al*.^4^. Thus, for 2011, Zn intake for Zimbabwe was reported as 12.3 mg person^−1^ day^−1^ based on maize grain Zn concentration of 28.0 mg Zn kg^−1^ and a maize supply of 300 g person^−1^ day^−1^. This represents 8.4 mg Zn person^−1^ day^−1^ derived from maize. Thus, if maize grain Zn concentration was 22.3 mg Zn kg^−1^, which is the mean value observed on the least productive fields in this study (Fig. 5), this would translate to 6.7 mg Zn capita^−1^ day^−1^. If maize grain Zn concentration was 25.2 mg Zn kg^−1^, which is the mean value observed on the most productive fields in this study (Fig. 5), this would translate to 7.6 mg Zn capita^−1^ day^−1^. It is therefore plausible that farmer management could improve nutrition, alongside other benefits of manure inputs to the soil. While a more detailed health economic analyses from variation in grain Zn due to farmer management could be conducted to guide policy decisions, Zn intake based on a predominantly maize diet is still unlikely to be sufficient under most conditions observed in the survey. Dietary diversification and biofortification interventions with improved crop varieties and micronutrient fertilizers are still likely to be needed to improve dietary Zn supply to sufficient levels for optimal health.

## Conclusions

Differences in agro-ecological region, soil nutrient status, crop type and farm-level SOM management drive substantial variation in Zn and Fe in staple diets. Therefore, the improvement of Zn and potentially Fe nutrition in crops lies not only on inherent soil properties but also on farmer management practices which influence SOM and N dynamics. This study provides insights for future interventions to promote better Zn and Fe nutrition in smallholder crop production systems, which could also include altered crop choice, use of biofortified crop varieties, and increased use of micronutrient fertilizers, together with wider strategies for dietary diversification.

## Supplementary information

Supplementary information

Dataset 1

## Data Availability

The datasets generated during and/or analysed during the current study are available from the corresponding author on reasonable request.
