# Supplementary material for: Fertilizer management and soil type influence grain zinc and iron concentration under contrasting smallholder cropping systems in Zimbabwe
Source: Sci Rep. 2019 Apr 23;9:6445. doi: 10.1038/s41598-019-42828-0 (PMC6478733; doi:10.1038/s41598-019-42828-0)
Supplement: Supplementary file 1 — Supplementary information [file 41598_2019_42828_MOESM1_ESM.pdf]

FERTILIZER MANAGEMENT AND SOIL TYPE INFLUENCE GRAIN ZINC AND IRON CONCENTRATION UNDER CONTRASTING SMALLHOLDER CROPPING SYSTEMS IN ZIMBABWE

Manzeke Muneta G[1,*], Mtambanengwe Florence[1], Watts Michael J[2], Hamilton Elliott M[2], Lark R. Murray[3], Broadley Martin R[3], Mapfumo Paul[1]

Supplementary material

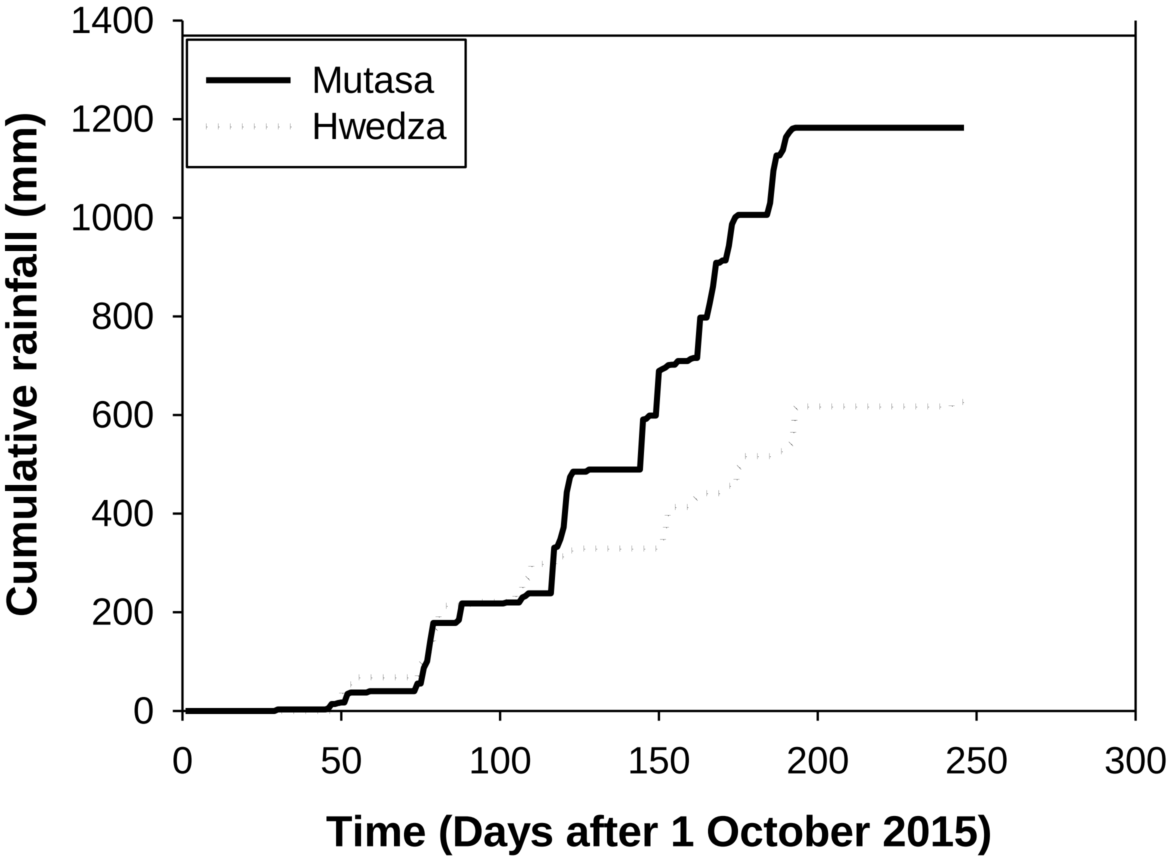

Figure 1: Cumulative rainfall received in Mutasa and Hwedza during the 2015/16 cropping season when crop samples were collected.

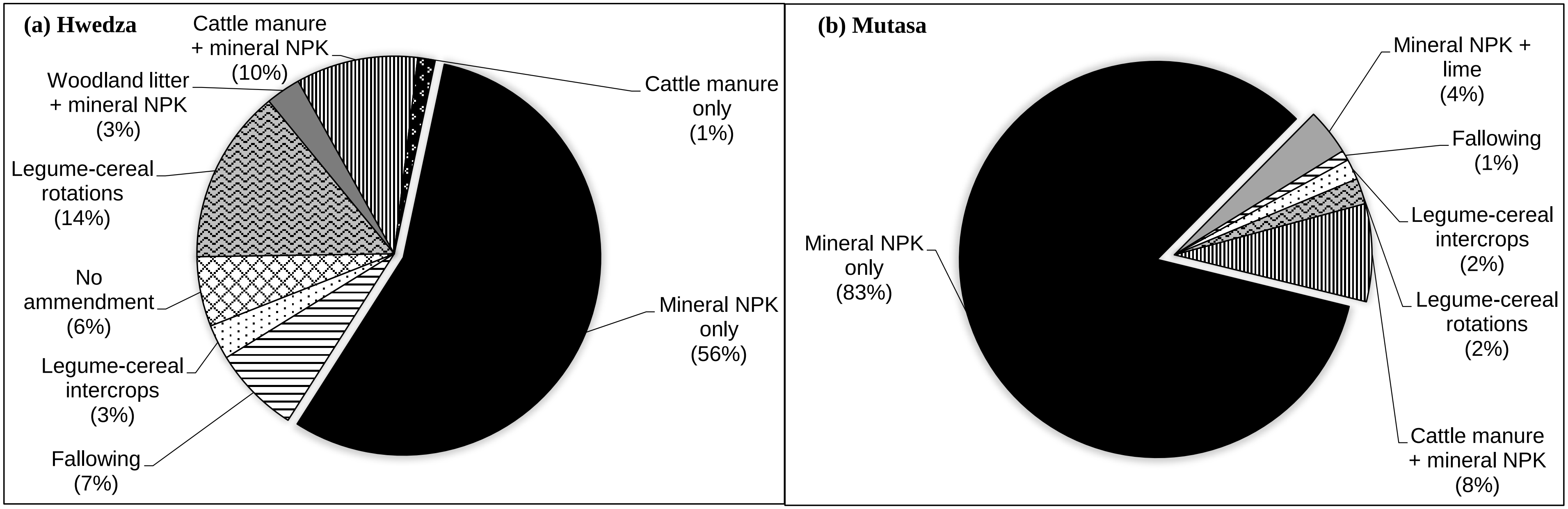

Figure 2: Farmer soil fertility management options employed in crop production in Hwedza and Mutasa Districts during the 2015-16 cropping season.

Table 1: Typical characteristics of "rich" and "poor" fields under smallholder cropping in Zimbabwe

Description	Fertilizer type applied	*Range of fertilizer rates applied (kg ha^{-1})
**a) Most productive field (MP)**[†]		
Soil fertility<br>High organic carbon averaging 0.7%. High soil $pH_{(CaCl2)}$ of up to 5.4. High content of exchangeable bases (calcium-Ca and magnesium-Mg), available P and effective cation exchange capacity (eCEC) of around 4.6 $cmol_{(c)}$ kg^{-1}. Soils are usually red or grey coloured with a relatively high clay content.<br><br>*Farmer management*<br>Most agronomic practises including planting and weeding are done on time. Crops on the majority of fields are fertilized with high amounts of mineral and/ or organic nutrient resources.<br><br>*Crops grown*<br>Often allocated to maize intercropped with field bean (*Phaseolus vulgaris*) or in rotation with groundnut<br><br>*Crop response to fertilization*<br>Fields with high crop growth and yield response to external fertilization (both organic and inorganic).<br><br>*Moisture content*<br>Higher moisture content due to better infiltration rates and water holding capacity facilitated by the high soil organic matter contents.	Farmers preferentially allocate organic nutrient resources (cattle manure, woodland leaf litter, compost) and high rates of mineral fertilizer. Quantities of fertilizer applied vary from farm to farm depending on which resource group the farmer belongs to (see Mtambanengwe and Mapfumo 2009).	20.0-172.5 kg mineral N ha^{-1} applied as compound D ($7N:14P_2O_5:7K_2O$) and ammonium nitrate (AN-34.5% N).<br><br>Upto 14 scotch carts[**] of cattle manure is often applied in combination with mineral fertilizer application.<br><br>*Typical maize grain yields attained*<br><br>0.8-5.0 t ha^{-1}
**b) Least productive field (LP)**[§]		
Soil fertility<br>Low organic carbon ranging from 0.3-0.4%. Low soil pH of around 4.5. Low exchangeable bases (Ca and Mg) and a low eCEC content averaging 2.9 $cmol_{(c)}$ kg^{-1}.	Sub-optimal rates of mineral fertilizer often applied to the maize crop, with no mineral	0-172.5 kg mineral N ha^{-1} applied as compound D

Light coloured with high sand content. Fields are often allocated to grain legume crops for soil fertility restoration.<br><br>*Farmer management*<br>Farmers do not often apply organic nutrient resources (e.g. compost, cattle manure, woodland leaf litter) and do not invest much labour and time towards production on these fields. Planting and/ or weeding is often done late, and fields are characterized by high weeds infestation due to infrequent weeding.<br><br>*Crops grown*<br>Often allocated to "*women crops*" such as groundnut, cowpea (see Mapfumo et al. 2001) and soybean. Small grains are also grown on such nutrient depleted soils.<br><br>*Crop response to fertilization*<br>Usually crop failure with no external fertilization. Low crop response to external addition of mineral and/ organic fertilizer. Crops have yellowish leaves, stunted and poorly established (uneven crop stand).<br><br>*Moisture content*<br>Low moisture retention resulting in sudden evidence of moisture stress in crops after a rainfall event.	fertilizer use on some of the fields. Organic nutrient resources are often not applied to these fields.	($7N:14P_2O_5:7K_2O$) and ammonium nitrate (AN-34.5% N).<br><br>Cattle manure is either not applied or applied after several cropping seasons at lower rates of about 2-5 scotch carts ha^{-1}.<br><br>*Typical maize grain yields attained*<br><br>0.1-0.8 t ha^{-1}

†, § Also referred to as "rich" and "poor" fields, respectively. * Rates from the soil and crop survey (this study). Range of fertilizer (organic and/ mineral) applied varied depending on farmer resource group. ** A scotch cart carries approximately 350-500 kg cattle manure load^{-1} ([31,34]). Typical characteristics of most and least productive fields drawn from Mtambanengwe and Mapfumo[32]; Masvaya et al.[35]; Vanlauwe et al.[36]; Zingore et al.[33;37].

Table 2: Grain yields and grain Zn and Fe concentration of sampled grains in both Hwedza and Mutasa District.

Crop type	Grain yields	Grain Zn concentration	Grain Fe concentration
	$t\ ha^{-1}$	$mg\ kg^{-1}$	
Maize (n=178)	0.1-5.2 (1.4 ± 0.05)	9-42 (24.0 ± 0.6)	8-66 (28.0 ± 0.6)
Cowpea (n=61)	0.04-1.0 (0.2 ± 0.04)	8-37 (22.5 ± 1.0)	18-108 (43.7 ± 1.9)
Sorghum (n=34)	0.1-1.4 (0.6 ± 0.2)	8-40 (22.9 ± 1.5)	16-308[†] (78.1± 9.6)
Finger millet (n=77)	0.05-1.5 (0.3± 0.09)	9-40 (24.9 ± 0.9)	25-139 (62.3 ± 2.9)

Figures in parentheses denote range ± standard error (SE). † = Possible dust contamination in one sorghum sample. Aluminium (Al) and vanadium (V) concentrations (used as indications of soil contamination-Joy et al.[41]) were 285 mg Al kg^{-1} and 0.50 mg V kg^{-1} for the sorghum sample.

Table 3: Descriptive statistics of soil Zn and Fe concentrations, soil pH and soil organic matter in Hwedza and Mutasa Districts, Zimbabwe

District	Locality: Ward Name	Statistics	Soil property: Total Zn concentration (mg kg^{-1})	Plant-available Zn concentration (mg kg^{-1})	Total Fe concentration (g kg^{-1})	Plant-available Fe concentration (mg kg^{-1})	SOM (%)	pH
1. Mutasa	**Mandeya**	Minimum	11.4	0.1	7.5	2.8	1.6	4.1
		Mean	35.5	0.6	39.4	16.6	6.0	4.7
		Median	31.8	0.4	30.5	15.1	6.0	4.6
		Maximum	97.9	9.2	166.1	40.5	11.4	6.2
	**Sahumani**	Minimum	6.3	0.1	4.5	1.8	0.7	4.0
		Mean	25.9	0.5	25.7	7.5	2.6	4.9
		Median	21.2	0.2	16.2	6.5	2.3	4.7
		Maximum	139.0	6.9	186.9	35.5	8.5	7.0
2. Hwedza	**Dendenyore**	Minimum	6.3	0.1	2.7	4.3	0.8	4.1
		Mean	43.2	0.9	41.3	14.8	3.0	4.9
		Median	18.6	0.7	15.4	14.9	2.1	4.7
		Maximum	193.3	2.5	125.3	36.9	7.5	6.7
	**Ushe**	Minimum	6.2	0.1	4.4	3.2	0.3	4.3
		Mean	12.1	0.4	7.6	11.9	0.9	5.1
		Median	11.2	0.3	6.7	9.7	1.0	4.8
		Maximum	23.3	1.5	13.0	33.0	1.6	7.3
**†F-test**			0.269ns	0.510ns	0.038*	0.141ns	<0.001***	0.008**

† F-test for significant differences between Districts. ns – not significantly different at P<0.05. *, **, *** significantly different at P<0.05, 0.01 and 0.001, respectively.
